# Active inspection with knowledge distillation for cost-effective fault prediction in manufacturing process

**DOI:** 10.1038/s41598-026-39412-8

**Published:** 2026-02-13

**Authors:** Junbong Heo, Minhyeok Son, Jaewoong Shim

**Affiliations:** 1 Department of Data Science, Seoul National University of Science and Technology, 232 Gongneung-ro, Seoul, Nowon-gu 01811 Republic of Korea; 2 Department of Industrial Engineering, Seoul National University of Science and Technology, 232 Gongneung-ro, Seoul, Nowon-gu 01811 Republic of Korea

**Keywords:** Fault prediction, Inspection cost, Knowledge distillation, Active inspection, Semiconductor manufacturing, Engineering, Mathematics and computing

## Abstract

Manufacturing processes involve various inspections aimed at identifying faulty products before they reach the final stages. These inspections are typically categorized into basic, conducted for all products, and advanced, conducted only for selected sampled products due to cost constraints. Recent advancements have leveraged inspection data to train machine learning models that predict potential faults in manufactured products. However, models using only basic inspection results, referred to as the basic model, often underperform compared to those that also use advanced inspection results, referred to as the advanced model, due to limited information. In this study, we propose a novel approach to train a basic model using knowledge distillation from an advanced model, achieving high prediction accuracy with reduced inspection costs. Additionally, we incorporate this distilled basic model into an active inspection framework during the inference phase to further improve the cost-effectiveness of inspections. Within this framework, the distilled basic model and the advanced model are selectively used, optimizing the usage of inspection costs. The effectiveness of our approach is demonstrated through a case study involving real-world data from a semiconductor manufacturer.

## Introduction

Fault prediction refers to the task of preemptively identifying potential faulty products in the manufacturing process. Despite numerous inspection steps designed to filter out faulty products, some faults may not be detected until the final shipment inspection, or worse, they may be discovered by customers after delivery. Late detection of faulty products leads to unnecessary continuation of the manufacturing process, incurring additional costs and potentially damaging the manufacturer’s reputation if faulty products reach customers. If potential faults could be detected early, it would be possible to rectify or discard the defective products before they cause further issues. To this end, there have been efforts to develop machine learning-based fault prediction models^1–7^. These models aim to use inspection results to identify defects early in the manufacturing process, thereby preventing costly outcomes.

Although ideally every product would undergo all types of inspections, the need for high productivity and reduced production costs often makes this impractical. Consequently, inspections are commonly conducted in a hierarchical manner. A set of low-cost, essential inspections is performed on all products to ensure broad coverage, while more costly and time-consuming inspections are applied only to a subset of products, typically through sampling. For clarity, we refer to these two inspection types as basic and advanced inspections, respectively.

Fault prediction models naturally reflect this inspection structure through their input variables. When only basic inspection results are available, fault prediction relies on a basic model that uses these inspection results as input variables. In contrast, for products that additionally undergo advanced inspections, an advanced model can be employed that incorporates both basic and advanced inspection results. Since the advanced model leverages a richer set of input variables, it generally achieves higher prediction accuracy, albeit at the expense of increased inspection cost. This distinction is used solely to describe differences in data availability arising from common inspection practices, rather than differences in model architecture.

**Fig. 1:**
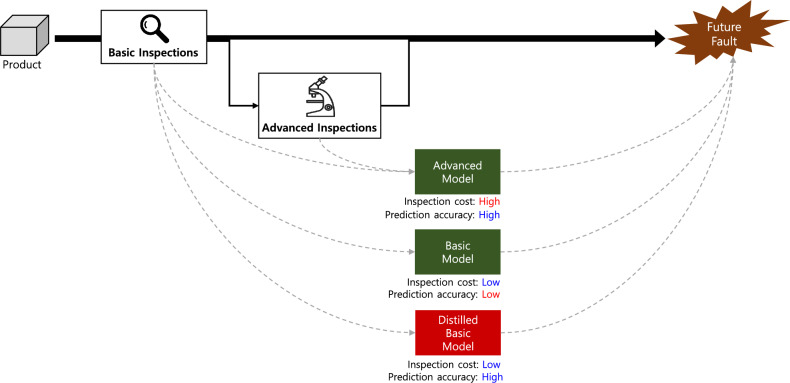
Problem situation and the goal of this study..

The situation addressed by our study is illustrated in Fig. 1. When operating the trained basic and advanced models, inspection results are required as input variables for inference, and these result in corresponding inspection costs. For a product to be evaluated by the advanced model, it must undergo both basic and advanced inspections, leading to higher inspection costs but potentially higher prediction performance. In contrast, the basic model only requires results from the basic inspection, resulting in lower inspection costs but likely lower prediction performance. The goal of this research is to achieve high fault prediction performance while incurring low inspection costs during the model operation phase. The existing active inspection framework^8^ achieves this goal by selectively applying basic and advanced models based on predictive uncertainty. Our research proposes a new method for training a basic model and incorporates it within the active inspection framework, further enhancing the cost-effectiveness of inspections for fault prediction accuracy.

Firstly, we propose a method to train an improved basic model utilizing knowledge distillation. Traditionally, knowledge distillation is a technique that transfers knowledge from a model with more complex architecture to a simpler one, primarily aiming to achieve high performance with reduced computational costs^9,10^. However, in this study, we employ knowledge distillation to achieve high performance with reduced inspection costs. Specifically, after the advanced model is trained, knowledge is transferred to the basic model during its training phase. Consequently, the trained basic model maintains low inspection costs required for operation while enhancing prediction performance. Furthermore, the basic model, improved through knowledge distillation, can be incorporated into an active inspection framework^8^ in operation phase. We investigate the effectiveness of the proposed method through a case study using a real-world dataset provided by a semiconductor manufacturer.

The main contribution of this study is to reinterpret knowledge distillation from the perspective of inspection cost reduction in manufacturing fault prediction. While conventional knowledge distillation primarily focuses on reducing computational complexity or model size, we apply it to transfer knowledge from a model that relies on high-cost inspection data to a model that operates only on low-cost inspection results, rather than introducing a new distillation algorithm. This formulation, combined with an active inspection framework, enables cost-effective fault prediction in practical manufacturing settings.

This paper is structured as follows. In Sect. “Related work”, we introduce related research. In Sect. “Proposed method”, we explain the proposed method leveraging knowledge distillation. In Sect. “Case study”, we present the experimental results of a case study. In Sect. “Conclusion”, we provide the conclusions of the paper.

## Related work

### Machine learning for fault prediction

Fault prediction within the manufacturing industry has significantly evolved, increasingly leveraging advanced machine learning techniques to enhance accuracy. Early methods primarily utilized statistical techniques and simple predictive models^11–13^. However, as computational resources expanded, these approaches have gradually evolved into more sophisticated machine learning techniques^14–16^. These modern approaches leverage a wide range of product-related information as input variables, such as process parameters, equipment sensor values, and inspection results. The output variable typically determines if a product will be identified as faulty at later stages, including during final shipment inspections or customer inspections^17^.

A broad spectrum of learning algorithms has been developed for fault prediction, ranging from basic classifiers such as logistic regression, decision trees, Bayesian classifiers, and k-nearest neighbors, to more robust ensemble models like random forests and gradient boosting^2,16,18,19^. Additionally, with the rise of deep learning, neural network models have increasingly been adopted for fault prediction^1,3,5–7^. These models excel at handling complex patterns and processing large datasets efficiently, making them particularly suitable for the intricate demands of modern manufacturing fault detection.

Recent studies have further advanced fault prediction by developing state-of-the-art learning frameworks and model architectures tailored to complex manufacturing environments^20–23^. While these efforts primarily aim to improve predictive performance through model design, they still depend on the availability of inspection data during both training and deployment. In practice, acquiring inspection results incurs substantial financial and temporal costs in manufacturing systems^24^. Practical budget constraints often limit the extent to which such data can be obtained. As a result, despite advances in fault prediction models, the high cost of inspection data remains a major obstacle to their widespread application.

To overcome these challenges, the active inspection framework has been proposed^8^. Given trained models, this framework improves the cost-effectiveness of inspection costs by selectively using basic and advanced models during the model’s operation phase. Building on this framework, we propose a training-stage approach based on knowledge distillation. The resulting distilled model can be seamlessly integrated into the active inspection framework during the model operation phase.

### Knowledge distillation

Knowledge distillation is a machine learning technique originally developed to address the challenges of deploying large, complex models on devices with limited computational power or environments requiring rapid inference^9,10^. The core principle of knowledge distillation involves transferring the knowledge from a large, sophisticated teacher model to a smaller, simpler student model. This method not only reduces the model size but also aims to retain the performance capabilities of the larger model, thus enabling the smaller model to deliver high accuracy predictions with reduced computational costs.

Various methods of knowledge distillation have been developed over time. The most widely used approach is the soft target method^10^, where the outputs probabilities of the teacher model are employed as soft targets for the student model, in addition to hard labels. Another technique is feature-based knowledge distillation, which utilizes the intermediate features or representations learned by the teacher to guide the training of the student model^25,26^. Additionally, relation-based distillation has emerged, focusing on replicating the relational aspects between data points captured by the teacher model^27^.

In the manufacturing industry, knowledge distillation has been applied to streamline the implementation of predictive models across various production aspects. These applications include predictive maintenance^28,29^, defect detection^30,31^, fault prediction^32,33^, and energy consumption prediction^34^. In these studies, knowledge distillation is primarily used to reduce model complexity or inference latency, enabling efficient deployment of machine learning models in resource-constrained or real-time manufacturing environments. By employing distilled models, manufacturers can deploy machine learning model directly onto the production floor while maintaining acceptable predictive performance.

Unlike traditional applications of knowledge distillation that primarily focus on computational efficiency, our study leverages this technique from a different perspective, namely, to reduce inspection costs associated with acquiring input variables. In manufacturing processes, the financial and temporal costs incurred by inspection procedures are typically substantial, rendering the computational cost of fault prediction models relatively negligible in comparison. Rather than compressing models for faster inference, we distill knowledge from an advanced model that relies on costly inspection data into a basic model that uses only low-cost inspection results. By employing this approach, we aim to achieve high prediction accuracy with reduced inspection costs, thereby boosting the practical utility of fault prediction in real-world manufacturing systems.

## Proposed method

### Problem statement

In this study, we develop a fault prediction model that predicts future faults of each product, utilizing a variety of inspection results as input variables. The inspection process in manufacturing can be divided into two categories: basic inspection and subsequent advanced inspection. Through the basic inspection, we acquire *n* basic inspection items $X_1^\text {basic}, X_2^\text {basic}, \ldots , X_n^\text {basic}$ for all products. On the contrary, we obtain *m* advanced inspection items $X_1^\text {adv}, X_2^\text {adv}, \ldots , X_m^\text {adv}$, which are only available for a select number of products due to the high cost of these inspections.

The fault prediction model outputs a binary variable indicating whether a product will ultimately be deemed faulty. Thus, the model essentially functions as a binary classifier. This binary classifier, for any given input variable value, outputs a probability estimate ranging from 0 to 1. This estimate is used as a criterion for determining whether a new product is normal or faulty, and is denoted as a fault score *p*.

Given that advanced inspection items are only available for a select number of products, two types of fault prediction models can be trained: the basic model $F^\text {basic}$ and the advanced model $F^\text {adv}$^8^. The basic model $F^\text {basic}$ predicts faults using only the basic inspection items as input variables. It can be represented as follows:

1 $$\begin{aligned} p^\text {basic} = F^\text {basic}(X_1^\text {basic}, X_2^\text {basic}, \ldots , X_n^\text {basic}) \end{aligned}$$

The advanced model $F^\text {adv}$ predicts faults using both basic and advanced inspection items. It can be represented as follows:

2 $$\begin{aligned} p^\text {adv} = F^\text {adv}(X_1^\text {basic}, X_2^\text {basic}, \ldots , X_n^\text {basic}, X_1^\text {adv}, X_2^\text {adv}, \ldots , X_m^\text {adv}) \end{aligned}$$

Naturally, the advanced model $F^\text {adv}$ is expected to outperform the basic model $F^\text {basic}$, as advanced inspection items can contain significant information crucial for fault prediction. The problem is that advanced model is not always available due to the cost of advanced inspection items.

The objective of this study is to maintain high fault prediction performance while minimizing the inspection costs when operating the models. During the model training phase, it is assumed that a complete training set, containing both basic and advanced inspection items, is available. First, a high-performance basic model is trained through knowledge distillation from the advanced model. Since the basic model does not rely on advanced inspection items as input variables, it incurs significantly lower inspection costs during operation. In the model operation phase, the improved basic model is incorporated into an active inspection framework, enabling further performance improvements in a cost-effective manner. The notations used to describe the proposed method are summarized in Table 1.

**Table 1:** Notations used in this paper.

Notation	Description
$F^\text {basic}$	The basic model
$F^\text {adv}$	The advanced model
$X_1^\text {basic}, X_2^\text {basic}, \ldots , X_n^\text {basic}$	Basic inspection items
$X_1^\text {adv}, X_2^\text {adv}, \ldots , X_m^\text {adv}$	Advanced inspection items
*m*	The number of the advanced inspection items
*n*	The number of the basic inspection items
$p^{\text {basic}}$	Fault score estimated by the basic model.
$p^{\text {adv}}$	Fault score estimated by the advanced model.
*y*	Target variable ; 0 for normal product, 1 for faulty product

### Training phase: knowledge distillation for cost-effective basic model

During the training phase, the advanced model $F^{\text {adv}}$ is first trained using the given training set, which includes basic and advanced inspection items $X_1^\text {basic}, \ldots , X_n^\text {basic}, X_1^\text {adv}, \ldots , X_m^\text {adv}$ as input variables and the fault status *y* as the output variable. Once the advanced model is trained, the basic model is subsequently trained.

**Fig. 2:**
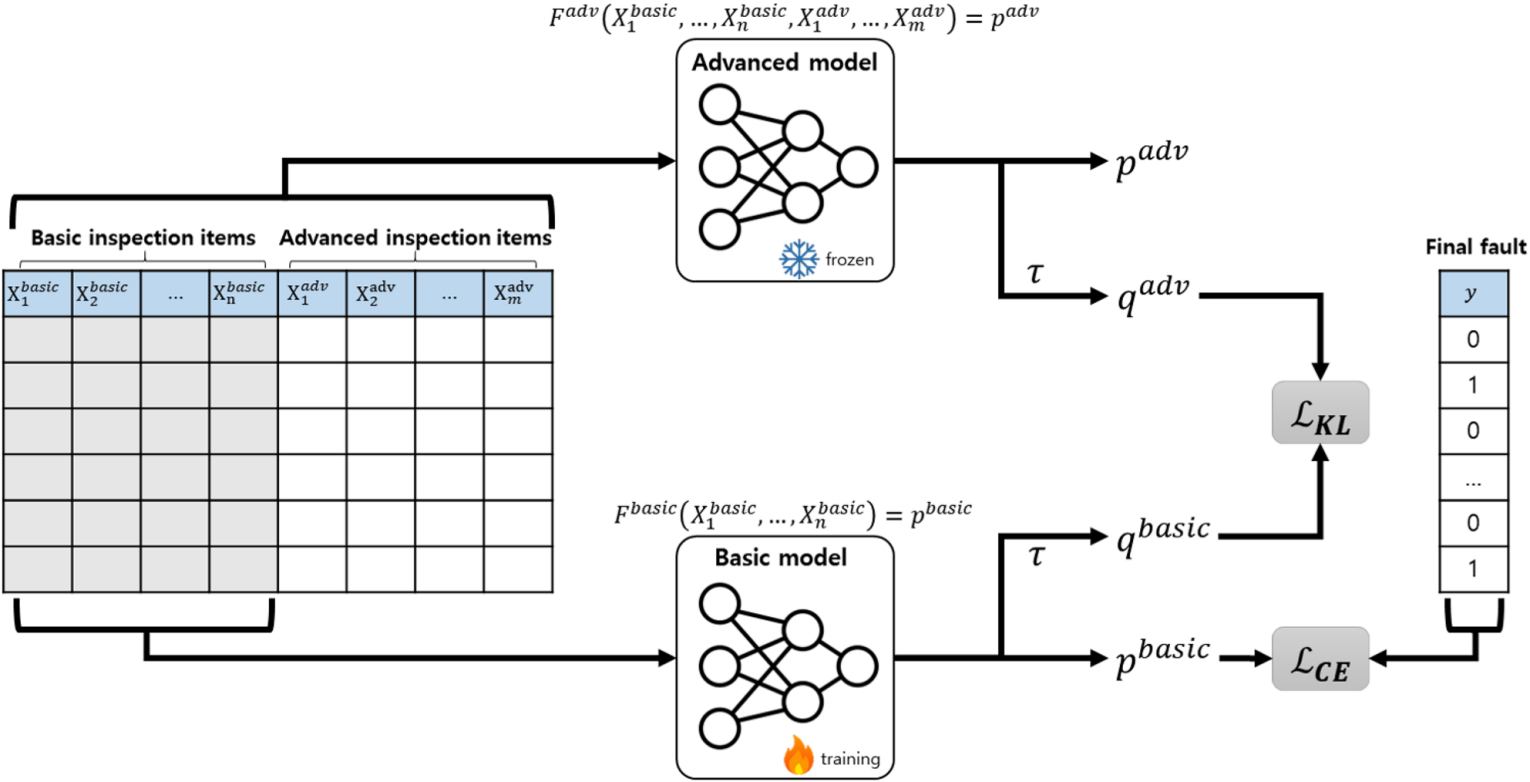
Illustrative description of the proposed method for training a basic model.

The procedure of training a basic model is described in Fig. 2. To create a high-performance basic model, we employ a knowledge distillation technique. While the original purpose of knowledge distillation is to transfer knowledge to a simpler model in terms of architecture, our goal is to transfer knowledge from a model with more input variables to one with fewer, specifically from the advanced model $F^{\text {adv}}$ to the basic model $F^{\text {basic}}$. Ultimately, the distilled basic model can achieve high performance without advanced inspection items.

When using neural networks as fault prediction models, the basic model $F^{\text {basic}}$ is typically trained with the binary cross-entropy loss $\mathscr {L}_{\textit{CE}}$:

3 $$\begin{aligned} \mathscr {L}_{\textit{CE}}=-y\log (p^{\text {basic}}) - (1-y)\log (1-p^{\text {basic}}) \end{aligned}$$

where $p^{\text {basic}}$ is the fault score predicted by the basic model $F^{\text {basic}}$. Specifically, $p^{\text {basic}}$ is calculated by applying a sigmoid function to the logit $z^{\text {basic}}$ from the last layer of the fault prediction model $F^{\text {basic}}$, as follows:

4 $$\begin{aligned} p^{\text {basic}} = \frac{1}{1+\exp (-z^{\text {basic}})} \end{aligned}$$

By minimizing $\mathscr {L}_{\textit{CE}}$, the output of basic model $p^{\text {basic}}$ can be closer to the ground-truth label *y*.

Given a trained advanced model $F^{\text {adv}}$, the Kullback-Leibler divergence loss $\mathscr {L}_{\textit{KL}}$ is introduced to utilize the knowledge of the advanced model in training the basic model $F^{\text {basic}}$. Here, the distillation temperature $\tau$ is introduced to derive the smoothed probabilities, $q^{\text {basic}}$ and $q^{\text {adv}}$ as follows:

5 $$\begin{aligned} q^{\text {basic}}= & \frac{1}{1+\exp (-z^{\text {basic}}/\tau )} \end{aligned}$$

6 $$\begin{aligned} q^{\text {adv}}= & \frac{1}{1+\exp (-z^{\text {adv}}/\tau )} \end{aligned}$$

where $\tau$ serves to adjust the level of smoothness. As $\tau$ increases, *q* becomes closer to 0.5. Then, $\mathscr {L}_{\textit{KL}}$ is calculated as follows:

7 $$\begin{aligned} \mathscr {L}_{\textit{KL}} = \tau ^2 \left\{ -q^{\textit{adv}} \log ( q^{\textit{basic}} ) - (1-q^{\textit{adv}}) \log ( 1-q^{\textit{basic}} ) \right\} \end{aligned}$$

Minimizing this loss reduces the difference between the outputs of the basic and advanced models, enabling the basic model to acquire knowledge by mimicking the advanced model. The total loss for training the basic model is expressed as a weighted sum of these two losses:

8 $$\begin{aligned} \mathscr {L}_{\textit{Total}}=(1-\alpha )\mathscr {L}_{\textit{CE}} + \alpha \mathscr {L}_{\textit{KL}} \end{aligned}$$

where $\alpha$ balances $\mathscr {L}_{\textit{CE}}$ and $\mathscr {L}_{\textit{KL}}$. By minimizing $\mathscr {L}_{\textit{Total}}$, we obtain the distilled basic model that achieve high performance using only the basic inspection items.

Additionally, we consider the use of non-neural network models as fault prediction models, $F^{\text {basic}}$ and $F^{\text {adv}}$. The concept of knowledge distillation is primarily applied to neural network models. However, for the tabular data we are currently dealing with, other machine learning models, such as random forests, still exhibit high performance^35^. Therefore, we also apply the concept of knowledge distillation to non-neural network models.

For the implementation of knowledge distillation in non-neural network models, the basic model is constructed as a regression model rather than a binary classification model^36^. This regression-based basic model is explicitly trained to estimate the output of the trained advanced model, $F^{\text {adv}}$, representing $p^\text {adv}$. The basic model is trained to minimize the mean squared error, $(p^\text {adv} - p^\text {basic})^2$, thereby aligning its outputs closely with the fault score from the advanced model.

### Operation phase: incorporation within active inspection framework

To enhance the cost-effectiveness of fault prediction, we incorporate the distilled basic model into the active inspection framework^8^. In this framework, the distilled basic model and the advanced model are selectively applied during operation based on prediction uncertainty.

**Fig. 3:**
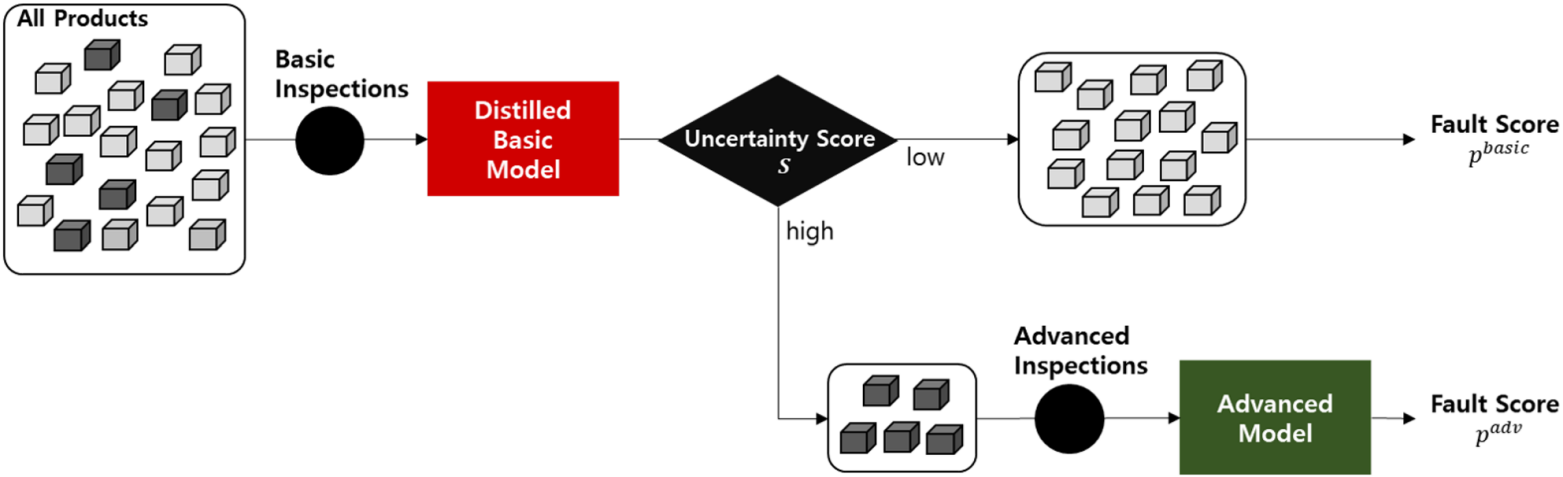
Active inspection with the distilled basic model.

As illustrated in Fig. 3, every product first undergoes basic inspections to collect basic inspection item values, $X_1^\text {basic}, X_2^\text {basic}, \ldots , X_n^\text {basic}$. Using these, the distilled basic model $F^\text {basic}$ computes an uncertainty score *S*, which reflects the model’s confidence in its prediction.

Products with high uncertainty scores then proceed to advanced inspections, where advanced inspection item values, $X_1^\text {adv}, X_2^\text {adv}, \ldots , X_m^\text {adv}$, are collected. For each product, the final prediction depends on the inspection pathway: if only basic inspections are performed, $F^\text {basic}$ produces $p^\text {basic}$; if both basic and advanced inspections are performed, $F^\text {adv}$ produces $p^\text {adv}$. This selective process ensures that advanced inspections are performed only when necessary, thereby improving overall cost-effectiveness.

## Case study

### Data description

**Table 2:** Dataset description.

Inspection recipe	Number of instances	Number of input variables	
		Basic inspection items	Advanced inspection items
Recipe1	18,471	8	91
Recipe2	245,006	8	80

To demonstrate the effectiveness of the proposed method, a case study was conducted using a real-world dataset provided by a semiconductor manufacturer in the Republic of Korea. The semiconductor manufacturing process consists of sequential phases: wafer fabrication, wafer test, assembly, and final test. After wafer fabrication, during the wafer test phase, electrical inspections are performed to obtain detailed electrical characteristics for each die on the wafer, including parameters such as current and voltage. Subsequently, in the assembly phase, the dies are manufactured into final products, which are then subjected to a final test to determine whether they pass or fail based on their functional integrity. The dataset used in this study includes inspection results from the wafer test phase as well as the final test outcomes, indicating whether each die passed or failed. Among the various inspections conducted during the wafer test phase, some are performed under severe conditions, making them time-consuming and costly. Therefore, these inspections are carried out only on a sampled subset of dies and are therefore categorized as advanced inspections. The remaining inspections, which are performed as full inspections across all dies, are categorized as basic inspections. The objective of this case study is to predict the failures in the final test based on the inspection results from the wafer test.

We utilized two datasets corresponding to different inspection recipes of wafer test, Recipe1 and Recipe2. Both recipes contain identical basic inspection items, but Recipe1 includes a greater number of advanced inspection items than Recipe2. Each product corresponds to an instance, and the total number of instances is higher in Recipe2. Each instance contains information about whether it is faulty or normal in the final test, represented as 1 or 0, with a fault rate of less than 1%. Inspection items serve as the input variables for the model, and the final test fault status is the output variable. A detailed description of the dataset is shown in Table 2.

### Experimental setting

We randomly divided the dataset into two parts: 50% for training and 50% for testing. The training set was used to train both the basic and advanced models, while the test set was utilized to evaluate the fault prediction performances.

For the fault prediction models, we employed two learning algorithms: neural network (NN) and random forest (RF), both of which have shown strong performance in prior work^8^. The NN model was configured with a single hidden layer comprising 10 neurons and utilized the ReLU activation function. The Adam optimizer was employed to update the parameters. We set aside a randomly selected 20% of the training set as a validation set, and the model that achieved the highest performance on this validation set was selected, with a cap at a maximum of 200 epochs. For knowledge distillation (KD) applied to the basic model, we set the $\tau$ at 2 and the $\alpha$ at 0.5. Regarding the RF models, each consisted of 500 trees, with each tree requiring a minimum of 10 instances for a split decision. For the advanced model and the basic model without KD, the RF classifier was employed using the Gini impurity as the splitting criterion. For the basic model trained with KD, an RF regressor was used, employing mean squared error as the splitting criterion.

In simulating the active inspection framework, we employed two methods for calculating the uncertainty score *S*: margin and biased margin, both of which were adopted from the original active inspection framework^8^.

- margin: This method calculates the inverse of the absolute difference between the fault score $p^\text {basic}$ and the normal score $1-p^\text {basic}$^37^:
  9 $$\begin{aligned} S = \frac{1}{|p^\text {basic} - 0.5|} \end{aligned}$$
- biased margin: This method modifies the margin technique to account for class imbalance. It assigns higher uncertainty to instances near the fault rate *FR*, which represents the percentage of faulty products in the training dataset^38^. It is calculated as:
  10 $$\begin{aligned} \begin{aligned} S&= {\left\{ \begin{array}{ll} \frac{p^\text {basic}}{FR}, & \text {if }p^\text {basic} \le FR;\\ \frac{1-p^\text {basic}}{1-FR}, & \text {otherwise}, \end{array}\right. } \end{aligned} \end{aligned}$$

Additionally, random sampling served as a baseline for comparison.

We defined advanced inspection rates as the proportion of products subjected to advanced inspections relative to the total number of products in the test set, reflecting the incurred inspection costs. We analyzed performance variations across different advanced inspection rates, which varied from zero to one in increments of 0.1. A rate of zero means that only basic inspections are performed on all products, whereas a rate of one indicates that every product undergo advanced inspections.

To summarize, the following approaches were compared in the experiments:

- *Random* Uses a basic model trained without KD, and selects instances for advanced inspection through random sampling without utilizing uncertainty scoring.
- *Margin* Uses a basic model trained without KD, applying the active inspection framework with margin as the uncertainty score.
- *BiasedMargin* Uses a basic model trained without KD, applying the active inspection framework with biased margin as the uncertainty score.
- *KD_Random* Uses a basic model trained with KD, and selects instances for advanced inspection through random sampling without utilizing uncertainty scoring.
- *KD_Margin* Uses a basic model trained with KD, applying the active inspection framework with margin as the uncertainty score.
- *KD_BiasedMargin* Uses a basic model trained with KD, applying the active inspection framework with biased margin as the uncertainty score.

The effectiveness of each method was assessed using the area under the receiver operating characteristic curve (AUROC), which measures the ability of a model to discriminate between faulty and normal products across all possible decision thresholds. An AUROC value of 0.5 corresponds to random guessing, while a value of 1.0 indicates perfect discrimination. AUROC is particularly suitable for fault prediction tasks with severe class imbalance, which is common in manufacturing systems. Each experiment was independently repeated 30 times, and the average results are presented.

### Results and discussion

The comparative results of the fault prediction models are displayed in Table 3. As shown in the table, for both Recipe1 and Recipe2 datasets, and employing NN and RF as the underlying models, the basic model trained with KD consistently outperformed the conventional basic model trained without KD. Additionally, the models trained with KD exhibited smaller standard deviations, suggesting more stable training outcomes. Notably, in the Recipe1 dataset, the basic model trained with KD even surpassed the performance of the advanced model. In the relatively smaller Recipe1 dataset, it is conjectured that KD acted as an effective regularizer, contributing to these results^39^.

**Table 3:** Comparison results of the fault prediction models in AUROC. (mean ± standard deviation).

		NN	RF
Recipe1	Basic model without KD	0.7097 ± 0.0574	0.7173 ± 0.0272
	Basic model with KD	**0.7346 ± 0.0527**	**0.7542 ± 0.0220**
	Advanced model	0.7327 ± 0.0485	0.7477 ± 0.0287
Recipe2	Basic model without KD	0.7282 ± 0.0101	0.6955 ± 0.0127
	Basic model with KD	**0.7297 ± 0.0085**	**0.7177 ± 0.0115**
	Advanced model	0.7374 ± 0.0106	0.7324 ± 0.0117

Bold values indicate the better performance between the basic models with and without KD.

**Fig. 4:**
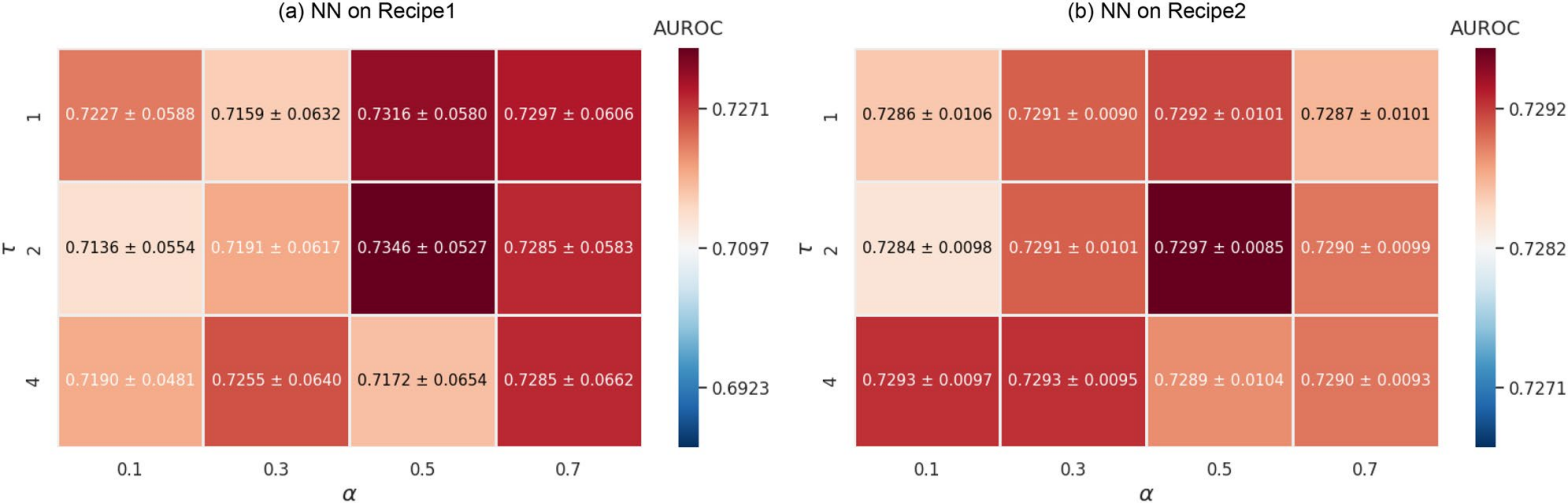
Sensitivity analysis of the hyperparameters $\tau$ and $\alpha$.

We conducted a sensitivity analysis on the hyparparameters $\tau$ and $\alpha$ for NNs on both Recipe1 and Recipe2. Figure 4 presents the results as heatmaps showing performance relative to the basic model without KD, with white indicating the baseline performance and red and blue representing performance improvements and degradations, respectively. As shown in the figure, the heatmaps are consistently dominated by red regions across all tested combinations of $\tau$ and $\alpha$, indicating performance improvements over the baseline for both datasets. These results indicate that the proposed approach is robust and not highly sensitive to the choice of $\tau$ and $\alpha$.

**Fig. 5:**
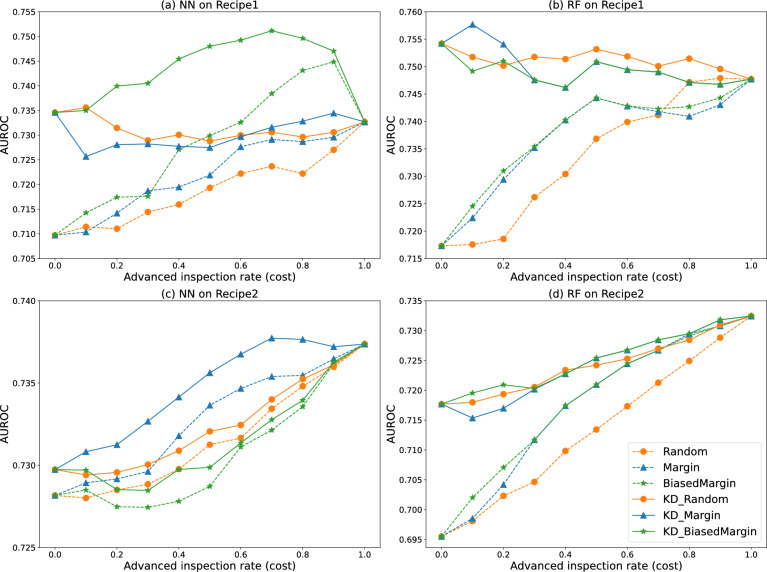
Comparison of average performance with active inspection framework.

The comparative results of the fault prediction models incorporated within the active inspection framework are depicted in Fig. 5. The x-axis represents the advanced inspection rate *r*, which can be directly interpreted as a proxy for inspection cost. Specifically, by normalizing the cost of a basic inspection to one unit and denoting the relative cost of an advanced inspection as $\kappa \gg 1$, the expected inspection cost per product can be expressed as $C = 1 + r \cdot \kappa$. Under this formulation, an x-axis value of 0 corresponds to performing only basic inspections for all products, whereas a value of 1 indicates that advanced inspections are conducted for all products. The y-axis denotes the fault prediction performance derived from the framework. Points closer to the top left therefore indicate higher prediction performance achieved with lower expected inspection cost, demonstrating superior cost-effectiveness.

The performances of the active inspection framework utilizing the conventional basic model are represented by dashed lines. For the **Random**, the fault prediction performance increased linearly with the advanced inspection rate. Meanwhile, **Margin** and **BiasedMargin**, which utilize the uncertainty score, demonstrated greater effectiveness in terms of inspection costs compared to **Random**, aligning with the results in the original paper^8^.

Solid lines represent the performance of the active inspection framework that incorporates the KD-trained basic model. The performance at an advanced inspection rate of 0 reflected the enhanced performance of the basic model due to KD, as previously confirmed in Table 3. This incorporation allowed **KD_Random**, **KD_Margin**, and **KD_BiasedMargin** to outperform **Random**, **Margin**, and **BiasedMargin** across all advanced inspection rates respectively. **KD_Random** exhibited a performance trend that interpolates between the basic and advanced model performances, similar to **Random**. Meanwhile, **KD_Margin** and **KD_BiasedMargin** displayed superior cost-effectiveness compared to **Random**. Specifically, in Recipe1 using NN (Fig. 5a), **KD_BiasedMargin** outperformed other baseline methods. Performance was better at advanced inspection rates ranging from 0.1 to 0.9 than at the extremes of 0 or 1, implying that selectively utilizing the basic model trained with KD and the advanced model can yield better results than exclusively using one model. In Recipe2 using NN (Fig. 5c), **KD_Margin** outperformed other methods. In Recipe1 using RF (Fig. 5b), **Margin** at an advanced inspection rate of 0.1 demonstrated the best performance, and in Recipe2 using RF (Fig. 5d), **BiasedMargin** showed superior performance on the whole. From an economic perspective, this improvement translates into a tangible reduction in expected inspection cost. For example, in Recipe2 using NN (Fig. 5c), when the relative cost of an advanced inspection is set to $\kappa = 10$, **KD_Margin** with an advanced inspection rate of $r = 0.4$ achieves comparable or superior AUROC to **Random** with $r = 0.7$, corresponding to a reduction of approximately $37.5\%$ in expected inspection cost.

Additionally, some results showed the active inspection framework performing worse in terms of cost-effectiveness than the random baselines, such as with **KD_BiasedMargin** in Recipe2 using NN. This may be attributed to the fact that, during knowledge distillation, the basic model can inherit not only useful knowledge but also overfitting tendencies or miscalibrated confidence from the advanced model, leading to less reliable uncertainty scores. These results suggest that the choice of uncertainty estimation method for the distilled basic model should be made carefully, taking into account dataset characteristics, such as severe class imbalance, and the underlying learning algorithm.

In summary, employing proposed KD during the training phase of the basic model has improved fault prediction performance without additional inspection costs. Further incorporating this enhanced model within the active inspection framework has shown improved cost-effectiveness in inspection. Although the proposed framework is evaluated using a semiconductor manufacturing dataset, it is not limited to this specific domain. The framework can be applied to other manufacturing or inspection-driven settings where inspection procedures incur heterogeneous costs and high-cost inspections are selectively performed. Such scenarios include non-destructive testing, battery cell testing, and quality inspection processes in electronics manufacturing. More generally, the proposed approach is applicable to systems that require cost-sensitive acquisition of additional inspection information.

## Conclusion

To accurately predict faulty products through a machine learning model, it is essential to conduct inspections to obtain values for input variables. Due to cost constraints, advanced inspections are typically limited to a subset of sampled products. In this study, we proposed a knowledge distillation-based method to train an enhanced basic model that does not require additional results from advanced inspections. By transferring knowledge from the advanced model to the basic model during the training process, we were able to improve the prediction performance of the basic model while maintaining low inspection costs. Incorporating this basic model into the active inspection framework further enhanced the cost-effectiveness of inspections for fault prediction accuracy. The effectiveness of this methodology was demonstrated through a case study using a real-world dataset provided by a semiconductor manufacturer.

For future work, we plan to explore various knowledge distillation techniques to train more cost-effective fault prediction models. Specifically, we plan to employ a teaching assistant model to bridge the information gap between advanced and basic inspections. Additionally, rather than a one-way transfer, we aim to facilitate mutual knowledge sharing between the basic and advanced models to leverage their combined strengths. Lastly, we intend to refine the sampling process of the active inspection framework, ensuring that the most appropriate models are used for each product considering the inspection budget.

## Data Availability

The datasets analysed during the current study are not publicly available due to the data privacy policies of the data-providing company, but are available from the corresponding author on reasonable request.
